# Targeting tumour necrosis factor alleviates signs and symptoms of inflammatory osteoarthritis of the knee

**DOI:** 10.1186/ar4044

**Published:** 2012-10-04

**Authors:** Walter P Maksymowych, Anthony S Russell, Peter Chiu, Alex Yan, Niall Jones, Tracey Clare, Robert GW Lambert

**Affiliations:** 1 Department of Medicine, University of Alberta, 562 Heritage Medical Research Building, Edmonton, Alberta, Canada T6G 2S2; 2 Department of Radiology, University of Alberta, 2A2.41 Walter Mackenzie Building, Edmonton, Alberta, Canada T6G 2B7

## Abstract

**Introduction:**

Inflammation associated with synovial expression of TNFα is a recognised feature of osteoarthritis (OA), although no studies have yet reported beneficial effects of anti-TNFα therapy on clinical manifestations of inflammation in OA.

**Methods:**

We conducted an open-label evaluation of adalimumab over 12 weeks in 20 patients with OA of the knee and evidence of effusion clinically. Inclusion criteria included daily knee pain for the month preceding study enrolment and a summed pain score of 125 to 400 mm visual analogue scale on the Western Ontario and McMaster University Osteoarthritis Index (WOMAC) pain subscale. The primary outcome was the Osteoarthritis Research Society International/Outcome Measures in Rheumatology Clinical Trials (OARSI/OMERACT) response criterion at week 12. Secondary outcomes included the WOMAC pain score 20% and 50% improvement, WOMAC stiffness and function scores, patient and physician global visual analogue scale, as well as target joint swelling.

**Results:**

Treatment was well tolerated and completed by 17 patients with withdrawals unrelated to lack of efficacy or adverse events. By intention to treat, an OARSI/OMERACT response was recorded in 14 (70%) patients. WOMAC pain 20% and 50% responses were recorded in 14 (70%) patients and eight (40%) patients, respectively. Significant improvement was observed in mean WOMAC pain, stiffness, function, physician and patient global, as well as target joint swelling at 12 weeks (*P *< 0.0001 for all). After treatment discontinuation, 16 patients were available for assessment at 22 weeks and OARSI/OMERACT response compared with baseline was still evident in 10 (50%) patients.

**Conclusion:**

Targeting TNFα may be of therapeutic benefit in OA and requires further evaluation in controlled trials.

**Trial registration:**

ClinicalTrials.gov: NCT00686439.

## Introduction

Osteoarthritis (OA) is the most common form of arthritis, involving approximately 10% of Canadians [1]. The disease is characterised by disruption of chondrocyte homeostasis with the balance being shifted toward tissue degradation, leading to the progressive loss of cartilage extracellular matrix. Although often considered a non-inflammatory arthropathy, the results of more recent studies indicate that cytokine and growth factor production is increased in all three joint components; namely, the synovial membrane, cartilage, and subchondral bone [2,3]. Among these factors, the cytokines TNF, IL-1, IL-6, and IL-17 seem most involved in the process of cartilage destruction [2,4], while cartilage repair that could restore the functional integrity of the joint is impaired because chondrocytes appear unable to respond to insulin-like growth factor-1 or respond abnormally to transforming growth factor-beta - growth factors that also modulate cytokine expression [2]. In addition, synovial inflammation is frequently evident in OA patients using magnetic resonance imaging (MRI) and has been associated with pain [5,6]. Arthroscopic studies suggest that localised proliferative and inflammatory changes of the synovium occur in up to 50% of OA patients, and the activated synovium may produce proteases and cytokines that accelerate progression of disease [7]. OA synovial macrophages exhibit an activated phenotype, as demonstrated by the production of both proinflammatory cytokines, such as IL-1 and TNF, and vascular endothelial growth factor [8-10].

To date, no specific therapy based on fundamental intracellular pathways of chondrocytes exists for the medical management of OA with the exception of anti-inflammatory corticosteroids and NSAIDs. However, our increased understanding of the molecular mechanisms underlying the degenerative process in OA has led to a possible targeted therapeutic approach to the management of the disease. Among the factors so far identified, TNF has received particular attention because of its predominance in the pathogenesis of other arthritic diseases. Results from one study showed that PEGylated soluble TNF receptor 1, an antagonist of TNF, inhibited both the increase in type II collagen cleavage by collagenase and the increase in glycosaminoglycan release observed in explant cultures of osteoarthritic articular cartilage [11]. Furthermore, PEGylated soluble TNF receptor 1, either alone or in combination with anakinra, an IL-1 receptor antagonist, frequently downregulated gene expression of matrix metalloproteases MMP1, MMP3 and MMP13 that are involved in cartilage extracellular matrix degradation. Conversely, PEGylated soluble TNF receptor 1 and anakinra upregulated aggrecan and type II collagen gene expression in about 50% of OA cartilage explant cultures. These findings suggest that inhibition of TNF may offer a useful therapeutic approach to the management of OA.

The present study was designed to provide preliminary evidence for the clinical efficacy and safety of the TNFα antagonist adalimumab in subjects with OA of the knee and inflammatory manifestations evident as joint effusion on clinical examination, whose pain persists despite maximum tolerated doses of conventional therapy.

## Materials and methods

### Patient population

This open-label trial of adalimumab was performed in patients ≥ 40 years of age with inflammatory OA of the knee. Patients had a diagnosis of OA of the index knee according to American College of Rheumatology criteria, including radiological evidence of OA (Kellgren-Lawrence grade 2 or grade 3) [12]. Inclusion criteria included continual pain for at least 6 months prior to inclusion in the study with daily knee pain in the month preceding study enrolment, and pain that persisted despite conventional treatment defined as any one of the following medications taken daily during any one of the preceding 6 months: acetaminophen (2 to 4 g/day), maximum tolerated and recommended doses of an NSAID, or acetaminophen/codeine combination (that is, Tylenol 2, 3, and 4;) taken at least three times daily. Patients had to have a summed pain score of 125 to 400 mm (visual analogue scale (VAS)) on the WOMAC pain subscale in the index (more symptomatic) knee, and the analgesic/NSAID dosage had to have been stable for 2 weeks prior to enrolment in the study, with concomitant use of NSAIDs permitted at stable doses during the study. Those patients receiving analgesic therapy were instructed to take stable doses in the 24 hours prior to each study assessment. Patients had to have clinical evidence of a knee effusion in the index (more symptomatic) knee at screening and at baseline. The study received local institutional review board approval from the University of Alberta Health Ethics Review Board and all patients provided written informed consent prior to participation.

Patients were excluded if they had evidence of an autoimmune cause of arthritis, clinically significant medical conditions including active infection, predominant patellofemoral disease, a concurrent medical or arthritic condition that could confound evaluation of the index joint (for example, post-traumatic or any secondary form of knee OA), a history of clinically significant trauma, surgery or diagnostic arthroscopy to the index knee, intra-articular injections of steroid and/or hyaluronate compounds into the index knee within 3 months of study enrolment, were in the upper tertile of the age-adjusted and race-adjusted norms for body mass index, and had a history of cancer or lymphoproliferative disease.

### Study procedures

Prior to study enrolment, each patient underwent a clinical evaluation including medical history, physical examination, laboratory studies, purified protein derivative skin test, chest X-ray, and a review of concurrent medications. Laboratory tests required for eligibility included a negative serum and urine pregnancy test, hepatitis B surface antigen, anti-hepatitis C antibody, and normal complete blood count, alanine aminotransferase, and serum creatinine. Patients received six biweekly subcutaneous injections of 40 mg adalimumab over 12 weeks. Patients were assessed at baseline and at weeks 4, 8, and 12 of treatment. Evaluations at each visit included the WOMAC (Likert 1 to 5 scale) [13], physician and patient assessments of global disease activity by 100 mm VAS score, target joint assessment for tenderness and swelling (0 to 3 scale for both), and self-reported expanded target joint assessment (1 to 5 scale) focused on impairment of activity and sleep [14]. All patients underwent a final efficacy and safety assessment at week 22.

### Outcome measures

The primary endpoint was the number (percentage) of patients achieving an Osteoarthritis Research Society International/Outcome Measures in Rheumatology Clinical Trials (OARSI/OMERACT) response at week 12 [15]. This is defined as an improvement in pain or function of at least 50% and a decrease of at least 20 mm on the VAS for pain or function, or occurrence of at least two of the following: decrease in pain of at least 20% and at least 10 mm on the VAS; improvement in function of at least 20% and a decrease of at least 10 mm on the VAS; and an increase in the patient's global assessment score by at least 20% and at least 10 mm on the VAS. Secondary endpoints included change in the WOMAC pain subscale score, WOMAC pain subscale 20% and 50% improvement, change in the WOMAC stiffness and function subscales, change in the patient and physician global assessment of disease status score, change in the target joint tenderness and swelling score, and change in self-reported activity and sleep impairment score from baseline to week 12.

### Statistical analysis

Because this was an uncontrolled open-label pilot study, analyses were primarily descriptive. Analysis was based on intention to treat. The proportion of patients achieving an OARSI/OMERACT response was calculated for each time point. Response was calculated according to an intent-to-treat analysis. Comparisons of each clinical measure were made between weeks 12 and baseline using Student *t *tests. A last-observation-carried-forward analysis was used for patients that did not complete the 12-week study.

## Results

### Patient population

The study population was typical of an OA population. No unexpected concomitant diseases, presenting baseline disease characteristics, or prior/concomitant therapies were noted. Almost all subjects were white Caucasians (19/20) except for one (categorised as other). There were nine males and 11 females of mean ± standard deviation (SD) (median, range) age 57.3 ± 9.2 (55, 46 to 77) years. Mean ± SD symptom duration was 4.7 ± 4.0 years, height was 168.8 ± 9.5 cm and weight was 83.5 ± 19.6 kg. Eleven patients had never smoked, five were ex-smokers, and four were current smokers. Nonsteroidal anti-inflammatory agents were taken by 15 patients at screening and baseline (diclofenac *n *= 5, ibuprofen *n *= 4, celecoxib *n *= 3, naproxen *n *= 3), acetaminophen by 14 patients, codeine by four patients, and tramadol by one patient. The target joint was the right knee for 12 patients and the left knee for eight patients. The Kellgren-Lawrence grade on radiography was grade 3 for 13 patients and grade 2 for seven patients. Eleven patients had previously had joint aspiration and intra-articular injection with corticosteroids. None of these had crystals detected. At screening, the mean ± SD summed WOMAC pain score was 299.5 ± 69.4 and the median (range) was 292 (171 to 445). The mean ± SD WOMAC pain subscale score was 59.9 ± 13.9 and the median (range) WOMAC pain score was 58.4 (34.2 to 89.0). A summary of disease duration and characteristics at baseline for all subjects treated with any dose of adalimumab is presented in Table 1.

**Table 1:** Baseline disease characteristics

Duration of osteoarthritis (years)	
Mean (standard deviation)	4.7 (4.0)
Median	2.5
Minimum-maximum	1 to 15
WOMAC pain (0 to 100)	
Mean (standard deviation)	60.78 (17.51)
Median	59.00
Minimum-maximum	28.80 to 88.20
WOMAC stiffness (0 to 100)	
Mean (standard deviation)	69.70 (16.27)
Median	75.00
Minimum-maximum	27.50 to 92.50
WOMAC function (0 to 100)	
Mean (standard deviation)	66.00 (19.36)
Median	65.50
Minimum-maximum	26.06 to 95.63
Patient global (0 to 100)	
Mean (standard deviation)	60.55 (21.96)
Median	61.00
Minimum-maximum	16.00 to 92.00
Joint tenderness (0 to 3)	
Mean (standard deviation)	1.75 (0.44)
Median	2.00
Minimum-maximum	1.00 to 2.00
Joint swelling (0 to 3)	
Mean (standard deviation)	2.15 (0.37)
Median	2.00
Minimum-maximum	2.00 to 3.00
Physician global (0 to 100)	
Mean (standard deviation)	69.65 (12.74)
Median	67.50
Minimum-maximum	46.00 to 95.00

WOMAC, Western Ontario and McMaster University Osteoarthritis Index.

### Clinical efficacy

#### Primary endpoint

At 12 weeks, 14 (70%) patients had achieved an OARSI/OMERACT response by intent-to-treat analysis. This response was observed at 4 and 8 weeks in nine (45%) patients and 14 (70%) patients, respectively. Treatment was discontinued at 12 weeks and patients were reassessed at week 22. Four patients were not available for the 22-week follow-up having discontinued from the study by 12 weeks. Two of these patients had an OARSI/OMERACT response at 12 weeks but had discontinued the study, due to a flare of interstitial colitis in one patient, after having received two doses of adalimumab, and the other relocating out of the country after having received all doses of adalimumab. A third patient moved away from the study site after having received two doses of adalimumab and had not achieved an OARSI/OMERACT response at 12 weeks. A fourth patient received only one dose of study drug and had not achieved an OARSI/OMERACT response at 12 weeks. Of the 16 patients who completed 12 weeks of treatment and follow-up there were only two patients who had a numerically worse WOMAC pain subscale score at week 12 compared with baseline, and in neither was the increase greater than 10%. An OARSI/OMERACT response was observed in 13 (65%) patients at 22 weeks. Of these, 10 (50%) patients had an OARSI/OMERACT response that was evident at both 12 and 22 weeks and three (15%) patients had an OARSI/OMERACT response at 22 weeks but not at 12 weeks. Two (10%) patients had an OARSI/OMERACT response at 12 weeks that was no longer evident at 22 weeks.

#### Secondary endpoints

A significant reduction was observed in WOMAC pain, stiffness, and function subscales at week 12 (Table 2). All three subscale scores were significantly lower at 8 weeks (*P *< 0.0001). The number (%) of patients who reported a reduction ≥ 20% and ≥ 50% in the WOMAC pain subscale score was 14 (70%) and eight (40%) using last-observation-carried-forward data.

**Table 2:** WOMAC subscales in patients with inflammatory osteoarthritis of the knee who received open-label adalimumab

	Baseline	4 weeks	8 weeks	12 weeks	*P *value^a^	22 weeks^b^
WOMAC pain	60.8 (17.5)	49.6 (21.0)	40.2 (23.4)	36.1 (23.6)	< 0.0001	41.5 (18.9)
WOMAC stiffness	69.7 (16.3)	58.5 (18.3)	44.1 (26.0)	37.0 (25.1)	< 0.0001	48.6 (20.1)
WOMAC function	66.0 (19.4)	53.2 (20.3)	41.1 (25.8)	39.2 (25.1)	< 0.0001	46.6 (21.3)

Mean (standard deviation) for Western Ontario and McMaster University Osteoarthritis Index (WOMAC) subscales over 22 weeks in 20 patients with inflammatory osteoarthritis of the knee who received open-label adalimumab for 12 weeks. ^a^Week 12 data versus baseline according to paired *t *test using last-observation-carried-forward data. ^b^Week 22 data are based on 16 patients who attended for assessment.

A significant reduction from baseline was observed in patient and physician global assessments at week 12 (*P *< 0.0001), and this was already evident by week 4 (*P *= 0.016 and *P *< 0.0001 for patient and physician global, respectively) and week 8 (*P *< 0.0001 for both) (Table 3).

**Table 3:** Secondary endpoints in patients with inflammatory osteoarthritis of the knee who received open-label adalimumab

	Baseline	4 weeks	8 weeks	12 weeks^a^	22 weeks^b^
Patient global	60.6 (22.0)	49.7 (22.1)**	37.4 (22.6)*	36.1 (21.9)*	44.4 (22.5)
Physician global	69.7 (12.7)	44.6 (17.8)*	34.5 (16.9)*	40.7 (22.0)*	40.1 (25.3)
Activity impairment	6.30 (1.17)	5.70 (1.53)	4.90 (1.59)**	5.10 (2.00)*	5.19 (1.80)
Sleep impairment	2.90 (0.64)	2.35 (0.81)	2.10 (0.72)**	2.05 (0.89)**	2.50 (0.73)
Target joint tenderness	1.75 (0.44)	1.00 (0.73)	0.95 (0.69)**	0.95 (0.94)**	0.81 (75)
Target joint swelling	2.15 (0.37)	1.45 (0.89)**	1.00 (1.03)*	1.15 (1.14)**	0.75 (0.77)

Mean (standard deviation) values for secondary endpoints over 22 weeks in 20 patients with inflammatory osteoarthritis of the knee who received open-label adalimumab for 12 weeks. ^a^Week 12 data versus baseline according to paired *t *test using last-observation-carried-forward data. ^b^Week 22 data are based on 16 patients who attended for assessment. *P < 0.0001, **P < 0.05.

A significant reduction from baseline was observed in self-reported activity at week 12 (*P *= 0.0025) (Table 3). This was already evident by 8 weeks (*P *= 0.0008). A significant reduction from baseline was observed in self-reported impairment of sleep at week 12 (*P *= 0.003) (Table 3). This was already evident by 8 weeks (*P *= 0.0008).

A significant reduction from baseline was observed in assessor evaluation of target joint tenderness at week 12 (*P *= 0.002) (Table 3). This was already evident by 8 weeks (*P *= 0.0004). A significant reduction from baseline was observed in assessor evaluation of target joint swelling at week 12 (*P *= 0.0005) (Table 3). This was already evident by 4 weeks (*P *= 0.0009) and 8 weeks (*P *< 0.0001). Of particular note, three patients had swelling evident at 12 weeks that was no longer evident on physical examination at 22 weeks (two patients with grade 2 swelling and one patient with grade 3 swelling).

### Safety

Adverse events were minor with no serious events being recorded. One patient required oral steroid for microscopic interstitial colitis after having received two doses of adalimumab. This was a pre-existing condition that was stable at baseline without requirement for any concomitant medication. The patient was withdrawn from the study at week 8.

## Discussion and conclusion

The results from this 12-week open-label study demonstrated that adalimumab seems effective in reducing the signs and symptoms of inflammatory OA of the knee. An OARSI/OMERACT response was observed in the majority (70%) of patients, and 40% of patients had a major response as defined by WOMAC pain subscale improvement ≥ 50%. All secondary variables also demonstrated significant improvement from baseline that was often observed by 8 weeks. The response was also maintained in most patients at the last time of follow-up at 22 weeks after treatment had been discontinued for 10 weeks. In a few patients the response was delayed and was not evident at 12 weeks but became apparent at 22 weeks. Of particular note, three patients had substantial swelling evident at 12 weeks that was no longer evident on physical examination at 22 weeks. This suggests that a period > 12 weeks may be preferable for a double-blind study.

The objective of this study was to obtain preliminary evidence that an anti-TNFα strategy might have clinical benefit in patients with clinically apparent inflammatory OA. Many patients were not taking maximum doses of NSAID and none were taking acetaminophen 4 g/day as recommended in treatment guidelines either because of lack of tolerability or because of lack of efficacy. The majority had also failed intra-articular steroids. We excluded patients who were in the upper tertile of the age-adjusted and race-adjusted norms for body mass index. Our patients therefore reflect a treatment-refractory group of patients and highlight the limited therapeutic options that are currently available. In cross-sectional studies of MRI in knee OA, knee effusions and synovitis have been shown to be more often present in persons with knee pain than in persons with a comparable amount of radiographic knee OA but without pain [16-19]. Synovial thickening seen on noncontrast-enhanced MRI in the infrapatellar region of knees with OA has shown low-grade synovial inflammation on biopsy and this feature was present in 73% of knees with early OA [20]. Follow-up of patients with symptomatic knee OA in the Boston Osteoarthritis of the Knee Study has shown that change in synovitis correlated with change in knee pain [21].

Other studies have also focused on targeting synovial inflammation in patients with knee OA for symptomatic relief. In addition to intra-articular steroids [22,23], one study suggested that intra-articular anakinra (IL-1 receptor antagonist) given to seven patients with knee OA resulted in improvement in pain that paralleled improvements in MRI synovial scores [24]. These studies, along with our observation that targeting anti-TNFα may be clinically beneficial, suggest that synovitis ought to be an important treatment target in patients with OA. A recent double-blind, placebo-controlled study could demonstrate no improvement from baseline to week 4 in knee OA symptoms after a single intra-articular injection of anakinra [25]. Serum anakinra concentrations were below the quantification limit 24 hours post dose for all patients, suggesting that there is no depot effect from intra-articular injection of anakinra and that repeat injection may be necessary.

To date there has been a paucity of studies on anti-TNFα agents in OA. Recently, however, one study reported findings of a pilot study of adalimumab in OA of the hands [26]. In this open-label study, 12 patients aged > 45 years with erosive/inflammatory OA of the hands defined by ≥ 2 tender and ≥ 2 swollen joints (distal interphalangeal, proximal interphalangeal, first carpometacarpal) despite NSAIDs were treated with adalimumab 40 mg every other week for 12 weeks. At the end of treatment, a statistically significant improvement in the number of swollen joints compared with baseline (*P *< 0.01) was observed. Although there were no statistically significant changes from baseline in the other efficacy measures, trends suggested modest improvements and individual patients had some benefit from treatment with adalimumab. Of more direct relevance to the present study is a published case report where adalimumab was used to treat severe OA of the knee [27]. The administration of adalimumab was associated with a decrease in nocturnal pain. After a second injection 2 weeks later, nocturnal pain in the patient's knee completely resolved. MRI analyses performed 6 months after initiation of a treatment regimen tailored to the patient's needs (40 mg every 3 to 6 weeks) indicated that adalimumab therapy decreased synovial effusions, synovitis and bone marrow oedema. This is of importance since the prevalence and size of bone marrow lesions have been shown to be predictive of cartilage damage [28]. In addition, bone marrow lesions have been associated with the severity of pain perceived by the patient [29,30]. Taken together, our data are consistent in suggesting that TNFα inhibition could significantly improve the condition of patients with severe OA particularly when accompanied by inflammatory features.

There were several limitations to our study. Since this was intended to be a proof-of-concept study, we were limited in sample size and an assessment of predictors of response was not feasible. We limited our analysis to a subgroup of patients with clinical evidence of inflammation that did not include the most overweight patients often seen in OA. Consequently, our results may not be generalisable to the broader category of patients with OA. The lack of a control group and an independent assessor hindered the ability to discriminate placebo effects from true therapeutic effects. Stable background use of NSAIDs was allowed and, as the dose was kept stable, should not have interfered with the clinical assessments. The duration of our study was short, and there was evidence in some patients that a longer course of treatment might have been beneficial, but we chose 3 months of treatment since responders to adalimumab in rheumatoid arthritis trials achieved a clinical response by 12 weeks. Our data showed that clinical responses were sustained in the majority of patients at 22 weeks, which was 10 weeks after treatment discontinuation. This suggests that future trials should also incorporate a treatment withdrawal design to determine whether a strategy of pulse dosing might be effective for symptomatic control. This is particularly relevant in view of the costs of treatment and the high frequency of OA in the population. The design of future trials will also need to address the high placebo response rates noted in previous studies, particularly those evaluating intra-articular therapies and glucosamine and chondroitin sulphate [25,31]. This may reflect expectation bias for invasive therapies or therapies already in widespread use and will require more complex study designs that include objective parameters of inflammation as well as cross-over designs.

In summary, short-term treatment with adalimumab for 12 weeks in patients with inflammatory OA of the knee refractory to conventional treatment resulted in clinical benefit in the majority of patients using the OARSI/OMERACT responder index. Major clinical improvement was seen in almost one-half of the patients that included improvement in function, daily activity, and sleep in addition to objective amelioration of joint effusion. A future long-term randomised, placebo-controlled study is now warranted and should examine the effects of treatment over a longer time frame and the sustainability of clinical response after treatment discontinuation.

## Abbreviations

IL: interleukin; MRI: magnetic resonance imaging; NSAID: nonsteroidal anti-inflammatory drug; OA: osteoarthritis; OARSI/OMERACT: Osteoarthritis Research Society International/Outcome Measures in Rheumatology Clinical trials; SD: standard deviation; TNF: tumour necrosis factor; VAS: visual analogue scale; WOMAC: Western Ontario and McMaster University Osteoarthritis Index.

## Competing interests

This study was funded by an unrestricted educational grant from Abbott (Abbott Park, IL, USA). WPM and RGWL have received research grants and/or consulting fees or other remuneration from Abbott.

## Authors' contributions

WPM designed and executed the study and prepared the manuscript. ASR, PC, AY, NJ, and TC executed the study, and reviewed study data and the manuscript. RGWL contributed to the study design, executed the study, and reviewed study data and the manuscript. All authors read and approved the final manuscript.
